# Unusual Presentation of Craniopharyngioma Pituitary Mass in a 71-Year-Old Female: A Case Report

**DOI:** 10.1155/2024/1333552

**Published:** 2024-10-04

**Authors:** Taylor F. Faust, Punuru Reddy, Jillian Weiss, Michael Steadman, Connie Morizio, Garrett Cail

**Affiliations:** ^1^Department of Research, Alabama College of Osteopathic Medicine, Dothan, Alabama, USA; ^2^Department of Internal Medicine, Decatur Morgan Hospital, Decatur, Alabama, USA

## Abstract

In this report, we present the case of a 71-year-old African-American woman experiencing 2 months of intermittent headaches and episodes of blurred vision. Despite a comprehensive medical history that revealed chronic conditions and previous unrelated surgeries, the initial evaluation appeared to be unremarkable. Following the discovery of a mass on an imaging and a subsequent biopsy, the diagnosis of craniopharyngioma (WHO grade I) was confirmed. However, a brain mass was identified after additional ophthalmologic examination and MRI. This case explores the significance of recognizing atypical presentations of a brain injury that required a specific approach for diagnosis, surgical intervention and treatment, and postoperative care. This case contributes to the constantly evolving understanding of atypical manifestations of tumor characteristics and their complexities, along with the need to develop appropriate patient management strategies and provide optimal outcomes.

## 1. Introduction

Craniopharyngioma is a rare benign calcified, solid, or solid–cystic tumor arising from the Rathke pouch remnants originating in the suprasellar region [1, 2]. This tumor has a bimodal distribution that affects children and adolescents aged 5–14 years and adults in the 5th through 7th decades, with the latter being more common [3]. There are two subtypes: adamantinomatous, which is more common in children, and papillary, which is seen almost exclusively in adults [4]. Symptoms of craniopharyngioma can vary, though the most notable include visual changes due to the optic chiasm being 10 mm superior to the sella turcica at the convergence of the optic fibers; blurred vision, which is generally the most common presenting symptom; and then peripheral vision field loss [5]. Additional symptoms can include increased intracranial pressure near the craniopharyngioma's surrounding anatomy, which can cause hormonal imbalances and headaches. The diagnosis of craniopharyngioma is often made by imaging, with computed tomography (CT) and magnetic resonance imaging (MRI) being the widely accepted standard.

Another tumor commonly found in the sellar region is pituitary adenoma [6]. It is a benign tumor of the pituitary gland formed from the ectoderm of the primitive floor of the mouth that migrates to the sella turcica, where it adheres to the neurohypophysis. Nonfunctioning pituitary macroadenoma is >1 cm, and incidence increases with age [7]. It does not produce hormonal or secretory symptoms nor respond to dopamine agonist treatment with dopamine agonists [7, 8]. Symptoms are usually the result of a mass effect: headache, visual disturbances, extraocular muscle palsy, and potentially hypopituitarism with large tumors [7]. A pituitary adenoma can be visualized with a T1-weighted MRI that shows a hypointense mass originating from the sella turcica [8, 9].

This report aims to document a rare presentation of craniopharyngioma from the sella turcica, which is outside the expected location for this disease process [1, 2]. It presents essential aspects of recognizing this disease process and managing postoperative complications. Most importantly, it challenges existing literature regarding the known locations of craniopharyngioma and suggests a need for further research.

## 2. Case Report

A 71-year-old African-American woman was evaluated after experiencing 2 months of intermittent headaches and episodes of blurred vision.

The patient's medical history includes allergic rhinitis, atypical chest pain, type 2 diabetes mellitus, essential hypertension, osteoarthritis, and Raynaud syndrome. Past surgical history includes bilateral cataract surgery, C6–C7 cervical vertebral fusion, and lumbar spine surgery. The patient is a nonsmoker and a nonalcohol drinker; she is married, has one child, and is now retired.

Patient medications included aspirin 325 mg daily, baclofen 10 mg twice a day, calcium 500 mg daily, diltiazem hydrochloride 120 mg twice a day, fish oil 1000 mg twice a day, losartan potassium 50 mg daily, gabapentin 300 mg three times a day, and tramadol–acetaminophen 37.5–325 mg three times a day. The patient's family history includes her mother, who died at 78 years old due to chronic obstructive lung disease, and her father, who died at 83 years old due to pneumonia.

Her blood pressure was 134/78 mmHg. She is 160 cm tall and weighs 67.5 kg, with a body mass index of 26.4 kg/m^2^. The pupils were equal and reactive to light, with intact extraocular muscles. The neck was supple without goiter or lymphadenopathy. The cardiac and respiratory sounds were regular. The abdomen was soft and nontender, with no signs of peritonitis. No deficits were observed in the nervous system.

Laboratory data can be seen in (Table 1).

The patient was evaluated by a retinal specialist for ongoing blurring of vision while reading with glasses. Visual acuity was 20/80 in both eyes. The ocular pressure was 17 mmHg on the right and 14 mmHg on the left, with normal ocular pressure ranging between 10 and 21 mmHg. The fundus examination revealed thinning of the macula, AV crossing changes, and no leaks. Bitemporal vision loss was noted. This pattern of vision loss combined with headaches was concerning for a potential brain mass and warranted an MRI study.

**Figure 1:**
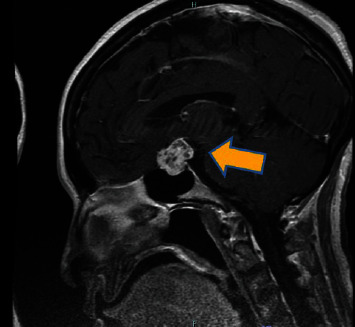
The sagittal T1-weighted image shows an inhomogeneous enhancing mass arising from the sella turcica, indicating a mass measuring 1.5 × 2.0 × 2.2 cm.

**Figure 2:**
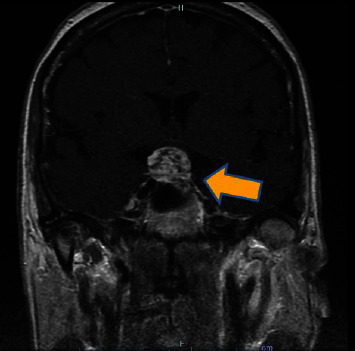
The T1-weighted coronal image shows an enhancing mass that invades the cavernous sinus, indicating a mass measuring 1.9 cm × 1.9 cm.

**Figure 3:**
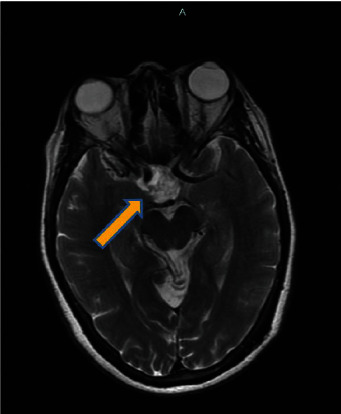
The axial T2-weighted image shows an enhanced mass measuring 1.9 cm × 2.1 cm with some cavernous sinus invasion.

An MRI of the brain with and without IV contrast was completed. The cerebrum and cerebral cortex findings featured an inhomogeneous enhancing mass in the pituitary fossa that extended into the suprasellar region with cavernous sinus involvement. The mass arose from the sella turcica and spread to the suprasellar cistern, partially displacing the hypothalamus, optic nerves, and optic chiasm superiorly. The imaging is shown below (Figures 1–3; see the mass measurements in the captions).

**Figure 4:**
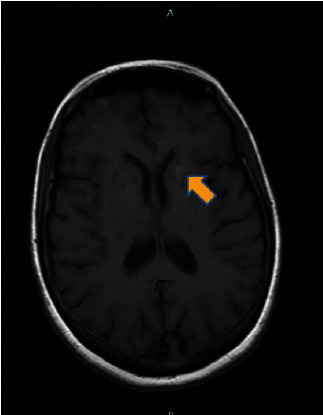
The axial T1-weighted image shows an enhancing nodule of the left caudate.

In addition to these findings, gadolinium was concentrated in the lateral portion of the left caudate head and the adjacent internal capsule, measuring less than 6 mm in dimension (Figure 4).

Imaging did not reveal midline shift, acute hemorrhage, or diffusion restriction, suggesting acute infarct. No other abnormal contrast enhancement was observed. A few areas of demyelination were present within the periventricular white matter, which was compatible with the renovated microvascular ischemic change.

The patient underwent a subsequent comprehensive laboratory workup on pituitary function to evaluate the tumor is found in (Table 2). This included adrenocorticotropic hormone (ACTH) levels, cortisol levels, follicle-stimulating hormone (FSH) levels, free T4, insulin-like growth factor 1 (IGF-1), luteinizing hormone (LH), prolactin, and thyroid-stimulating hormone (TSH). Due to the patient being through menopause, the reference ranges for LH and FSH can be variable as there is no established standard. All laboratory results obtained were found to be within the normal range. The complete pituitary laboratory function work up can be seen in (Table 2).

Based on the information provided, this patient was diagnosed with a pituitary mass extending into the suprasellar region and potentially impacting adjacent structures such as the optic nerves and chiasm. This provided context regarding her history of intermittent headaches and episodes of blurry vision. Given her age and presentation, a differential diagnosis could include a benign macroadenoma, such as a nonfunctioning pituitary adenoma or other sellar and parasellar lesions, including lymphoma, sarcoidosis, histiocytosis X, and metastasis. The differential diagnosis of the lesion in the left caudate head and adjacent internal capsule lesion also included lymphoma and granulomatous diseases of sarcoidosis and histiocytosis.

Laboratory workup of the patient, including hormone levels within the normal range, suggests that the mass may not secrete excess hormones. This would rule out functioning pituitary adenomas such as prolactinomas, growth hormone-secreting adenomas (causing acromegaly), and ACTH-secreting adenomas (causing Cushing's disease). The plasma cortisol levels were determined to be below the normal range, which was addressed by surgical intervention shortly after the laboratory results became available.

A transsphenoidal approach to resection is standard for many pituitary lesions, as it allows direct access to the sella turcica with minimal disruption to the surrounding brain tissue. Pathological examination of the biopsy will be critical to determining the nature of the tumor, including whether it is a benign adenoma, a more aggressive pituitary carcinoma, or another type of lesion entirely.

The patient was referred to neurosurgery, where she underwent transsphenoidal resection of the sellar/suprasellar mass, which is considered standard for resection of this type of mass [2]. A biopsy of the mass was obtained and sent to pathology for intraoperative consultation. The surgical case was concluded without further incident following this resection and biopsy. The pathologist reviewed the biopsy, described as a 1.0 cm × 0.89 cm × 0.3 cm aggregate of tan-pink to red soft tissue, which confirmed the diagnosis of craniopharyngioma (WHO grade I).

Postoperatively, the patient's headaches and episodes of blurred vision were entirely resolved. However, the postoperative course was complicated by a triphasic response with diabetes insipidus (DI) followed by the syndrome of inappropriate secretion of the antidiuretic hormone (SIADH) in which the sodium levels were decreased to 109.0 mmol/L (136–145 mmol/L), suggesting recurrent DI. The diagnosis of panhypopituitarism was also made. Following surgical resection of the tumor, she was treated with hydrocortisone 20 mg in the morning and 10 mg in the evening, desmopressin acetate 0.1 mg half a tablet once a day, and levothyroxine 50 µg daily to manage these findings.

A follow-up laboratory workup was completed 6 months after these interventions (Table 3) and was notable for a decrease in plasma cortisol at 0.7 µg/dL (5–25 µg/dL).

Close follow-up after surgery was suggested to manage the complications mentioned above and monitor the possible recurrence of the tumor. Furthermore, the participation of ophthalmic and endocrine specialists was deemed necessary to manage her visual symptoms and further hormonal dysfunctions comprehensively. Additionally, although small, the finding of a focus on gadolinium enhancement in the lateral portion of the left caudate head and adjacent internal capsule was suggested to be followed by a specialist, as it could represent an unrelated pathological process in the brain.

Regarding the patient's other medical issues, the continuous management of type 2 diabetes, essential hypertension, and other chronic conditions is vital for overall healthcare management.

The patient's initial clinical evaluation, which included the unremarkable physical exam without focal neurologic deficits, could initially have obscured the urgency or presence of the sizable pituitary mass found on the MRI. However, her symptoms of headaches and blurry vision were key signs that pointed to a neurological cause, particularly involving the sellar region.

Recovery and future treatment will depend on pathological results, the extent of resection achieved, and patient response postoperatively. Serial imaging studies might be ordered to monitor the area of resection, and additional treatments, including medical therapy or radiation therapy, could be considered depending on the final diagnosis.

After undergoing tumor resection and experiencing the postsurgical complication of DI, the patient's condition has significantly improved. As of publication, the symptoms of blurred vision and headaches reported during the initial encounter have entirely resolved.

## 3. Discussion

This report discusses the case of a 71-year-old African-American woman who exhibited two neurological symptoms—intermittent headaches for 2 months and blurred vision. Subsequent investigation revealed an enhancement of mass organization from the sella turcica within the pituitary fossa, extending into the suprasellar region and involving the cavernous sinus. Pathological investigation identified the mass as craniopharyngioma. These tumors are generally recognized for their occurrence in the suprasellar region and are classified as intracranial epithelial neoplasms, which arise from the craniopharyngeal duct or the Rathke pouch [10]. In this report, the typical characteristics of craniopharyngiomas were not observed, highlighting a unique case that deviates from what is commonly seen in the existing literature today. For example, the tumor in the case of our patient originated in the sellar region, a departure from the expected origin of pituitary adenomas [11]. This intriguing aspect of the case is related to the tumor's location due to the well-documented history of the literature on the origin of craniopharyngiomas. At the time of submission and to the authors' best knowledge, this case is the first to report a craniopharyngioma that arises from the sella turcica.

There are two primary categories of craniopharyngioma—adamantinomatous and papillary. Adamantinomatous craniopharyngiomas, associated with mutations in the CTNNB1 gene encoding beta-catenin, can be identified by their histological appearance of the palisading peripheral columnar epithelium and stellate reticulum that appear as the enamel pulp of developing teeth [12]. Papillary craniopharyngiomas, associated with mutations in the CTNNB1 gene encoding beta-catenin, are generally seen in middle to late adulthood [13, 14]. Although papillary craniopharyngiomas are more commonly seen in adult populations, adamantinomatous craniopharyngiomas are more common overall [13, 14]. Unlike adamantinomatous craniopharyngiomas, there are no calcifications and “wet keratin” on histological and radiographic analysis in papillary craniopharyngiomas [15].

Craniopharyngiomas are most commonly seen in the suprasellar region and arise from the pituitary stalk. Less commonly, they can be seen within the sella turcica itself [16] and even more rare within the third ventricle or on the elements of the optic system [14, 15]. Craniopharyngiomas can be diagnosed by CT, which gives a clear picture of a distinguishing feature of calcifications. MRI can also be a useful diagnostic tool that visualizes both cystic and solid forms of the tumor and hypothalamic involvement [17, 18]. These growths are not homogeneous, with solid, cystic, and calcified components [19]. Symptoms of this tumor can vary. Initially, >50% of patients present with headaches and visual deficits (peripheral vision loss). These symptoms are consistent with the patient presented in this case report. About 40%–87% of patients have >1 hormone deficit, specifically 17%–27% with endocrine symptoms such as diabetes and hypothalamic dysfunctions such as overeating and disturbed sleep. Other documented symptoms include blood pressure, irregular heart rate, and temperature dysregulation [2, 17, 20].

Pituitary adenomas are usually located in the sella turcica or the sphenoid sinus [21]. These adenomas can expand and disrupt the framework. Adenomas can be somatotrophs that produce other hormones, such as growth hormone and prolactin. These are most seen in young patients who lead to acromegaly and gigantism in childhood [22]. In older adults, they usually have acromegaly without gigantism [23]. They are also capable of producing thyroid hormone, causing symptoms similar to hyperthyroidism [24, 25]. Lactotroph adenoma, otherwise known as prolactinoma, is another hormone-producing tumor and the most common form of pituitary adenoma, [4] clinically presenting with galactorrhea in women [26]. A corticotroph adenoma is an ACTH-producing mass that results in excess cortisol, leading to Cushing disease [11]. Gonadotroph adenoma leads to increased levels of LH and FSH. Furthermore, thyrotroph adenomas, due to TSH, can also occur and present with hyperthyroidism [11]. These features of pituitary adenomas were absent in the patient presented in this report, providing additional evidence for the diagnosis of this particular tumor in our patient.

The principal treatment option for this type of tumor is surgical excision with external beam radiation generally used in cases of refractory tumor growth [2]. Other treatment options include intracystic radiation, bleomycin therapy, or other systemic chemotherapy regimens, though their clinical efficacy is undetermined compared to the mainstay resection treatment [2]. This type of tumor should be considered in any patient with a history of bitemporal vision loss with headache or evolving headache, as in the case of our patient. However, a variety of symptoms could be present [2]. MRI with and without contrast is the definitive imaging option of choice [1]. Laboratory studies, including CBC, CMP, and hormone studies, should be ordered to monitor the disease's severity and any dangerous levels that could influence immediate management [3].

## 4. Conclusions

This case report provides a unique presentation of craniopharyngioma originating in sella turcica, a departure from the typical origin of the suprasellar region. The patient's symptoms presented as intermittent multimonth headaches and blurred vision, with a lack of other notable clinical findings. The patient was successfully treated with transsphenoidal resection of the tumor, the preferred surgical intervention for tumors of this region. The distinct location of this tumor challenges the existing literature and suggests further research into craniopharyngioma origins and other tumors outside of the suprasellar region.

Further research to understand the origins of craniopharyngiomas and other tumors of the sellar region can provide valuable information for diagnostic purposes, symptomatology treatment planning, and understanding the varied potential impacts on surrounding structures. Knowing the tumor's location in the healthcare process offers a precise path to a more personalized approach to patient treatment and care.

## Figures and Tables

**Table 1:** Laboratory data during the initial workup.

Laboratory data	Lab values	Reference ranges
Chemistry		
Sodium	138.0 mmol/L	(136–145 mmol/L)
Potassium	4.6 mmol/L	(3.5–5.1 mmol/L)
BUN	15.0 mm/dL	(8–22 mg/dL)
Creatinine	0.7 mg/dL	(0.5–0.9 mg/dL)
Glucose	75.0 mg/dL	(70–104 mg/dL)
Calcium	9.4 mg/dL	(8.8–10.2 mg/dL)
TSH	1.36 µU/mL	(0.27–4.2 µU/mL)
Free T4	0.85 µg/dL	(4.5–11.2 µg/dL)
Hematology		
Hemoglobin	13.3 g/dL	(12.0–16.0 g/dL)
Hemoglobin A1c	5.90%	(≤6%)
WBC	6.7 × 1000/µL	(4.8–10.8 s× 1000/µL)
Hematocrit	40%	(37.0–47.0%)
Platelets	308.0 × 1000/µL	(130–400 × 1000/µL)

**Table 2:** Laboratory workup on complete pituitary function. Note that because the patient is postmenopausal, there are no reliable reference ranges for LH and FSH.

Pituitary function work up	Lab values	Reference ranges
TSH	1.3 µU/mL	(0.35–4.93 µU/mL)
Free T4	1.04 µg/dL	(0.7–1.48 µg/dL)
ACTH	26.0 pg/mL	(ND–46 pg/mL)
Plasma cortisol	3.0 µg/dL	(5 to 25 µg/dL)
LH	15.0 mUI/mL	^*∗*^
FSH	38.4 mUI/mL	^*∗*^
Prolactin	7.2 ng/mL	(3.46–19.40 ng/mL)
IGF-1	17.3 µg/dL	(5–25 µg/dL)

^*∗*^Due to the patient being through menopause, the reference ranges for LH and FSH can be variable as there is no established standard.

**Table 3:** Laboratory data during the postoperative workup.

Laboratory data	Lab values	Reference ranges
Chemistry		
Sodium	144.6 mmol/L	(136–145 mmol/L)
Potassium	4.6 mmol/L	(3.5–5.1 mmol/L)
Chloride	107.3 mmol/L	(98–107 mmol/L)
BUN/creatine ratio	13.8	(5–20)
BUN	11.0 mm/dL	(8–22 mg/dL)
Creatinine	0.8 mg/dL	(0.5–0.9 mg/dL)
Glucose	93.0 mg/dL	(70–104 mg/dL)
Calcium	9.5 mg/dL	(8.8–10.2 mg/dL)
TSH	0.012 µlU/mL	(0.27–4.2 µIU/ml)
Free T4	1.15 µg/dL	(4.5–11.2 µg/dL)
Plasma cortisol	0.7 µg/dL	(5–25 µg/dL)
Hematology		
Hemoglobin	13.0	(12.0–16.0 g/dL)
Hemoglobin A1c	5.70%	(≤6%)
WBC	10.7 × 1000/µL	(4.8–10.8 × 1000/µL)
Hematocrit	38.9%	(37.0%–47.0%)
Platelets	376.0 × 1000/µL	(130–400 × 1000/µL)

## Data Availability

The data used to support this study's findings are available from the corresponding author upon reasonable request.

## References

[1] DeAngelis L. M., Wen P. Y., Loscalzo J., Fauci A., Kasper D., Hauser S., Longo D., Jameson J. L. (2022). Primary and Metastatic Tumors of the Nervous System. *Harrison’s Principles of Internal Medicine*.

[2] Karavitaki N., Cudlip S., Adams C. B. T., Wass J. A. H. (2006). Craniopharyngiomas. *Endocrine Reviews*.

[3] Jensterle M., Jazbinsek S., Bosnjak R. (2019). Advances in the Management of Craniopharyngioma in Children and Adults. *Radiology and Oncology*.

[4] Larkin S. J., Ansorge O. (2013). Pathology and Pathogenesis of Craniopharyngiomas. *Pituitary*.

[5] Ireland A. C., Carter I. B. (2023). Neuroanatomy. *Optic Chiasm*.

[6] Kleinschmidt-DeMasters B. K. (2016). Histological Features of Pituitary Adenomas and Sellar Region Masses. *Current Opinion in Endocrinology, Diabetes & Obesity*.

[7] Fitzgerald P. A., Papadakis M. A., McPhee S. J., Rabow M. W., McQuaid K. R., Gandhi M. (2024). Nonfunctioning Pituitary Adenomas. *Current Medical Diagnosis & Treatment*.

[8] Melmed S., Jameson J. L., Loscalzo J., Fauci A., Kasper D., Hauser S., Longo D., Jameson J. L. (2022). Pituitary Tumor Syndromes. *Harrison’s Principles of Internal Medicine*.

[9] Herse P. (2014). Pituitary Macroadenoma: A Case Report and Review. *Clinical and Experimental Optometry*.

[10] Prabhu V. C., Brown H. G. (2005). The Pathogenesis of Craniopharyngiomas. *Child’s Nervous System*.

[11] Kobalka P. J., Huntoon K., Becker A. P. (2021). Neuropathology of Pituitary Adenomas and Sellar Lesions. *Neurosurgery*.

[12] Sekine S., Takata T., Shibata T. (2004). Expression of Enamel Proteins and LEF1 in Adamantinomatous Craniopharyngioma: Evidence for Its Odontogenic Epithelial Differentiation. *Histopathology*.

[13] Zacharia B. E., Bruce S. S., Goldstein H., Malone H. R., Neugut A. I., Bruce J. N. (2012). Incidence, Treatment and Survival of Patients With Craniopharyngioma in the Surveillance, Epidemiology and End Results Program. *Neuro-Oncology*.

[14] Crotty T. B., Scheithauer B. W., Young W. F. (1995). Papillary Craniopharyngioma: A Clinicopathological Study of 48 Cases. *Journal of Neurosurgery*.

[15] Sartoretti-Schefer S., Wichmann W., Aguzzi A., Valavanis A. (1997). MR Differentiation of Adamantinous and Squamous-Papillary Craniopharyngiomas. *AJNR. American Journal of Neuroradiology*.

[16] Price E. B., Moss H. E. (2014). Osborn’s Brain: Imaging, Pathology, and Anatomy. *Neuro-Ophthalmology*.

[17] Müller H. L. (2014). Craniopharyngioma. *Endocrine Reviews*.

[18] Lara-Velazquez M., Mehkri Y., Panther E. (2022). Current Advances in the Management of Adult Craniopharyngiomas. *Current Oncology*.

[19] Lee I. H., Zan E., Bell W. R., Burger P. C., Sung H., Yousem D. M. (2016). Craniopharyngiomas: Radiological Differentiation of Two Types. *Journal of Korean Neurosurgical Society*.

[20] Karavitaki N., Brufani C., Warner J. T. (2005). Craniopharyngiomas in Children and Adults: Systematic Analysis of 121 Cases With Long-Term Follow-Up. *Clinical Endocrinology*.

[21] Thompson L. D. R., Seethala R. R., Müller S. (2012). Ectopic Sphenoid Sinus Pituitary Adenoma (ESSPA) With Normal Anterior Pituitary Gland: A Clinicopathologic and Immunophenotypic Study of 32 Cases With a Comprehensive Review of the English Literature. *Head and Neck Pathology*.

[22] Akirov A., Asa S. L., Amer L., Shimon I., Ezzat S. (2019). The Clinicopathological Spectrum of Acromegaly. *Journal of Clinical Medicine*.

[23] Minematsu T., Miyai S., Kajiya H. (2005). Recent Progress in Studies of Pituitary Tumor Pathogenesis. *Endocrine*.

[24] Lopes M. B. S. (2017). World Health Organization Classification of Tumors of the Pituitary Gland: A Summary. *Acta Neuropathologica*.

[25] Pereira B. D., Raimundo L., Mete O., Oliveira A., Portugal J., Asa S. L. (2016). Monomorphous Plurihormonal Pituitary Adenoma of Pit-1 Lineage in a Giant Adolescent With Central Hyperthyroidism. *Endocrine Pathology*.

[26] Ellison D., Love S., Chimelli L., Harding B. N., Lowe J., Vinters H. V. (2012). *Neuropathology E-Book: A Reference Text of CNS Pathology*.

